# ETIOLOGICAL APPROACH TO CHRONIC URTICARIA

**DOI:** 10.4103/0019-5154.60348

**Published:** 2010

**Authors:** D S Krupa Shankar, Mukesh Ramnane, Eliz Aryal Rajouria

**Affiliations:** *From the Department of Dermatology, Venereology and Leprosy, Manipal Hospital, Bangalore, Karnataka, India.*

**Keywords:** *Auto antibodies*, *autoimmune disease*, *autologous serum skin test*, *CIU*, *skin prick test*

## Abstract

**Background:**

In 1769, William Cullen introduced the word “urticaria” (transient edematous papules, plaque with itching). Urticaria affects 15-25% of people at least once in their life time. It is a clinical reaction pattern triggered by many factors causing the liberation of vasoactive substances such as histamine, prostaglandins and kinins. Urticaria is classified according to its duration into acute (< 6 weeks duration) and chronic (>6 weeks duration). Various clinical investigations may be initiated to diagnosis the cause.

**Aims:**

To evaluate the types of chronic urticaria with reference to etiology from history and investigations.

**Materials and Methods:**

A total of 150 patients with chronic urticaria of more than six weeks were studied. Autologous serum skin test (ASST) was performed after physical urticarias were excluded. Standard batteries of tests were performed after ASST in all patients; and other specific investigations were done where necessary. Skin prick test was done in idiopathic urticaria.

**Results:**

The study sample consisted of 62 male and 88 female patients with a mean age of 21-40 years. About 50% of patients showed an ASST positive reaction, 3.9% were positive for antinuclear antibody (ANA), IgE titer was elevated in 37%, *H. pylori* antibodies was positive in 26.7%. Thyroid antibodies were positive in 6.2%. Giardia and entamoeba histolytica was reported in 3.3% on routine stool examination and on urinalysis 8% had elevated WBC counts; 12% showed para nasal sinusitis, with maxillary sinusitis of 7.3%. Random blood sugar was high in 5.3%. Four patients had ASOM, two had positive KOH mount for dermatophytes, abdominal USG showed cholecystitis in two patients. Recurrent tonsillitis was noted in two patients. Urticaria following intake of NSAIDs was observed in four patients and with oral contraceptive pills in one patient. Contact urticaria to condom (latex) was seen in one patient. Cholinergic (4.7%) and dermographic (4.7%) urticaria were the predominant physical urticarias. Prick test was performed in idiopathic urticaria with maximum reactions to food antigens (25%) where brinjal was the commonest, 9% to dust in which spider web was the most common, 8% to pollen where parthenium and amaranthus were the commonest, followed by *A. flavus* in fungi, pigeon in epithelia and cockroach in insects.

**Conclusion:**

Nearly half of the patients had chronic autoimmune urticaria on the basis of ASST. A significant number of them had serological makers of autoimmune activity. ASST provides an easy, inexpensive investigation in CU and helps direct attention to underlying systemic auto immune diseases. The presence of these auto antibodies was significantly associated with more frequent and longer lasting urticarial attacks. Exhaustive work ups with extensive laboratory diagnostics, challenge tests, and prick testing should be reserved for individual cases following detailed history.

## Introduction

In 1769, William Cullen introduced the word “urticaria” (transient edematous papule, plaque with itching). Urticaria affects 15-25% of people at least once in their life time.[1] It is a clinical reaction pattern triggered by many factors causing the liberation of vasoactive substances such as histamine, prostaglandins and kinins.[2] Clinically, uritcaria is classified into acute (duration <6 weeks) and chronic (duration >6 weeks) type.[3] Etiologically, urticaria is classified broadly into immunological mediated (IgE dependent and non-IgE dependent) and non-immunological mediated. After physical exertion as well as mechanical, thermal or electromagnetic stimuli (cold, heat, pressure, water, UV light) various forms of physical urticaria can occur due to non specific mast cells activation.[2] In the etiology based classification of urticaria, the idiopathic form is where no cause is found and is the most frequent, constituting up to 70% of cases.[2] About 30-50% of patients with chronic idiopathic urticaria have circulating histamine releasing auto antibodies to the high affinity IgE receptor FcεRI on the basophil and mast cells, or less commonly, antibodies to IgE.[4,5] The term autoimmune urticaria is increasingly being accepted for this subgroup of patients.[4] The autologous serum skin test (ASST) as defined by Sabroe *et al.*[6] is currently the best *in vivo* clinical test for detection of *in vitro* basophil histamine releasing activity with 65-71% of sensitivity and 78-81% specificity.

The diagnostic approach to urticaria is very broad and includes a comprehensive clinical, chemical and serological laboratory work-up. Prick test is performed for suspected foods, food additives along with allergy to dust, mites, fungi and epithelia. Challenge test is used to identify triggers in physical urticaria.

## Materials and Methods

A prospective observational study of 150 patients including 62 male and 88 female between the age of 10-70 years; with CU was conducted in the department of dermatology and venereology at Manipal Hospital, Bangalore. Clinical details of all patients were recorded using a standard proforma and an informed consent was taken from those who underwent investigations [Table 1]. Selected patients were subjected to a physical challenge test and laboratory investigations based on his/her history including: X-ray of para-nasal sinuses prick test with a panel of common food allergen, dust, mites, pollen, fungi.

**Table 1:** Test performed in patient of CU

Complete blood count, Random blood sugar, Erythrocyte sedimentation rate, total eosinophil count, peripheral blood smear
Routine stool and urine examination
LFT, T3, T 4, TSH
Immunoglobulin, IgE
ASST, ANA, skin prick test
X-ray para nasal sinus
Antibody for *H-pylori* and thyroid
Complement, Anti-streptolysin-O titre
Challenge test

### Methodology for autologous serum skin test

**Figure 1:**
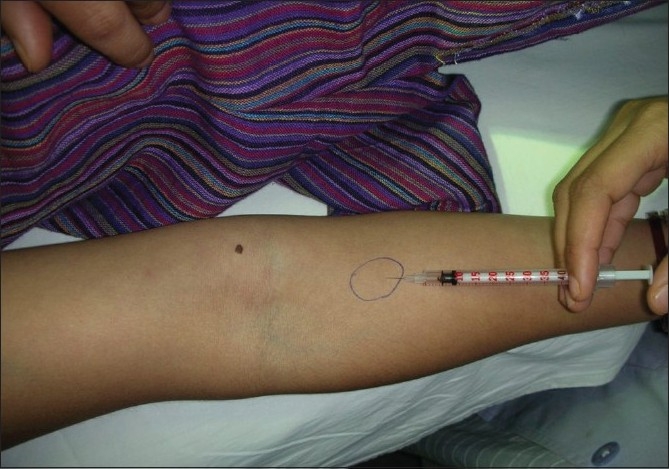
Method of performing and site for autologous serum skin test

**Figure 2:**
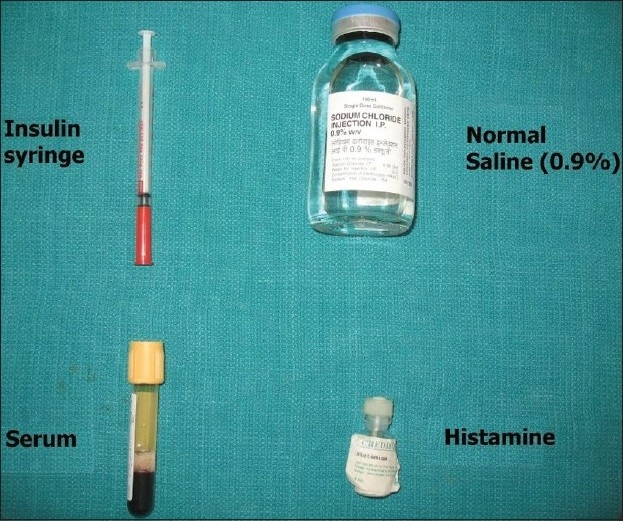
Easily available materials for autologous serum skin test

ASST was performed as follows: About 5 ml venous blood was collected in a sterile vacutainer and allowed to clot at room temperature for 30 minutes. Serum was sent to the clinical laboratory for centrifugation at 2000 rpm for 15 minutes and 0.05 ml of autologous serum (test) was then injected intradermally using a 1 ml insulin syringe (30 gauge needle) to the right forearm 2 cm below the cubital fossa and similarly 0.05ml of 0.9% sterile normal saline (control) was injected intradermally to the left forearm [Figures 1 and 2]. 0.05 ml of histamine (10 μg/ml) was injected into volar aspect of the left forearm as a positive control. A serum induced erythematous wheal with a diameter of 1.5 mm more than the saline induced response within 30 minutes was taken as a positive test. In all subjects, antihistamine and steroids were withdrawn at least 72 hours and one week prior to skin test, respectively.[7]

### Methodology for prick test

**Figure 3:**
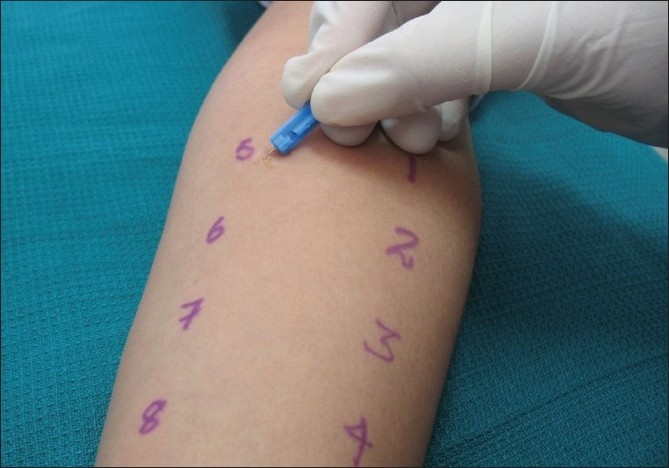
Procedure for performing prick test

Prick test was performed in patients with idiopathic urticaria with history of allergic reactions to food, dust and mites to trace out Type I hypersensitivity reaction (IgE mediated). Detailed history regarding antigens was recorded in the proforma, and consent was obtained. Emergency drugs (for anaphylaxis) were kept ready before starting the procedure. The preferred test sites are the back and forearms.[8] Histamine solution (H) was used as a positive control and a buffer solution (S) was used as a negative control. Readings were taken after 30 minutes and 60 minutes. Positive reactions manifest in the form of erythema, wheals, papules and vesicles. Positive controls should show reactions and negative controls should not show any reaction. Using the help of a dropper, the antigens are placed 3 cm apart and are number according to the present code. Using a lancet a superficial prick is made 45 degrees tangentially to the skin surface, with out bleeding or pain [Figure 3].

### Interpretation of prick test

A wheal of 3 mm diameter with surrounding edema can be recorded as a positive reaction.

**Table:** 

<2 mm	Negative
2-3 mm	Positive/Negative
3-5 mm	+
>6 mm	+ +
>6 mm with pseudopodia	+ + +
>6 mm with vesicles	+ + + +

Exclusion criteria in this study were

1) urticaria less then 6 weeks, 2) patients taking antihistamines or steroids or any other medications, 3) pregnant or lactating women, 4) severely ill patients and immuno-compromised patients, 5) patients with irregular follow up.

### Statistical methods

Descriptive statistical analysis has been carried out. Chi- square and Fisher Exact test have been used to find the significant association of ASST with the ANA, Thyroid- antibodies, IgE and *Helicobacter pylori*.

## Result

The male:female ratio among all urticaria subgroups was about 1:1.3. The average age in all urticaria categories belonged to the reproductive age groups (20-40years). Patients <20 years old show mostly infective focus.

**Graph 1:**
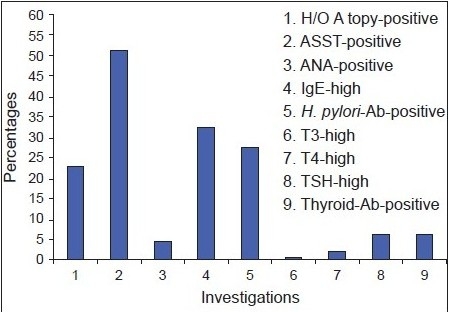
Relevant investigations for chronic urticaria

ASST was performed in all patients of CU after excluding physical urticaria. 65/127 patients who underwent ASST showed a positive reaction (51.1%). *H. pylori* antibodies were significantly higher (46.2%) for ASST positive patients when compared to ASST negative patients, which show significant *P* value (<0.001) [Table 2 and Graph 1]. Similarly, antinuclear antibodies (ANA) were positive in 7.7% of patients who were ASST positive, which showed moderately significant *P* value (0.026). On further investigation for autoimmune diseases systemic lupus erythematosus (SLE) was diagnosed in two patients. Thyroid antibodies were present in 10.8% of patients with positive ASST, which showed moderately significant for *P* value (0.034). IgE was elevated in only 35% of patients with ASST positive which is not significant.

**Table 2:** Association of autologous serum skin test positive and negative with antinuclear antibody, Thyroid -Ab, IgE, *H. pylori*

Factors	ASST				*P* value
		
	Negative (n = 62)		Positive (n = 65)		
			
	No	%	No	%	
ANA					
Negative	62	100.0	60	92.3	0.026*
Positive	0	0.0	5	7.7	
Thyroid-Ab					
Negative	61	98.4	58	89.2	0.034*
Positive	1	1.6	7	10.8	
IgE					
Negative	42	67.7	42	64.6	0.710
Positive	20	32.3	23	35.3	
*H. Pylori*-Ab					
Negative	58	93.5	35	53.8	<0.001**
Positive	4	6.5	30	46.2	

* moderately significant

** strongly significant

Giardia and *E. histolytica* were present in 3.3% of patients on routine stool examination. On analysis of urine 8% of patients showed a high WBC count. Random blood sugar levels were significantly elevated in 5.3% of patients, and they were started on anti diabetic therapy following further investigations. Gingitivitis with dental caries was observed in 10 patients. Acute suppurative otitis media (ASOM) was present in four children with history of ear discharge. Dermatophytes were present in KOH mount from two patients and antifungal therapy was given. The patients responded well. Throat pain with enlarged, inflamed tonsils was noted in two patients.

In two patients with abdominal pain following fatty food intake, cholecystitis was diagnosed based on the abdominal ultrasonogram report; 7.3% of patients showed maxillary sinusitis followed by frontal sinusitis in 2% and ethmoidal sinusitis in 2.7%. IgE was significantly high in patients with ethmoidal sinusitis compared to sinusitis at other sites [Table 3].

**Table 3:** Routine screening tests

Giardia, *E. histolytica* in stool routine-5 patients
*H. pylori* in stool routine-1 patient
High WBC count in urinalysis-12 patients
ASOM-4 patients
Tonsillitis-2 patients
Dermatophytes with positive KOH mount-2 patients
Dental caries-10 patients
Random blood sugar-2 patients
Paranasal sinusitis-18 patients
Cholecystitis-2 patients

**Graph 2:**
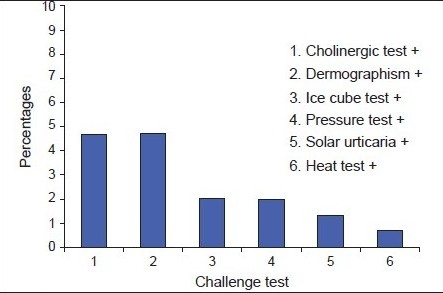
Physical urticaria

Challenge tests [Graph 2] were performed in 23 patients with clinical features of physical urticaria. 4.7% of patients showed cholinergic urticaria, 4.7% showed symptomatic dermographism, 2% showed cold urticaria with ice cube test, 10.2% showed delayed pressure urticaria, 1.3% showed with solar urticaria and 0.7% of patients showed localized heat Urticaria.

Skin Prick test was done in 22 patients who had idiopathic urticaria, six patients had a strongly suggestive history of food and dusts induced aggravation and were subjected to prick testing, out of which 10 were males and 18 females. Among them 60.7% patients showed reactions to more than five antigens, and maximum reaction was seen for foods, followed by dust, pollen, mites, fungi, epithelia and lastly insect [Table 4], and three patients did not show any reaction.

**Table 4:** Skin prick test

Food	Brinjal > Rice > Chili > Milk > Banana > Ginger
Dust	House dust > Spider web > Cotton dust
Pollen	Parthenium > Amaranthus > Cassica > Magnifera
Insect	Cockroaches > Ant > Mosquitos > House fly

## Discussion

Chronic Urticaria is a common disease that can persist or recur over long periods of time spanning years causing distress and at times being life threatening. As a cause is often not found, it presents the physicians with a diagnostic and therapeutic challenge.

To evaluate the role of a comprehensive diagnostic approach, a prospective evaluation of 150 patients was done over a period of one year. The youngest patient was a one-year-old and the oldest was 70, with mean ages of 25- 40 years. Eighty eight patients were male. Male to female ratio was 1:1.3. The most frequent areas of involvement were arm > back > legs. There was no significant difference between patients with or without auto antibodies regarding sites.

There is now growing evidence that 30-50% of CIU results from an autoimmune process involving functional histamine-releasing anti FcεRI, auto antibodies or less commonly, anti IgE-autoantibodies.[9] As patients with autoimmune antibodies have no distinctive diagnostic clinical features, current clinical diagnosis depends on autologous serum skin test. A positive test is suggestive but not diagnostic of an autoimmune basis.[10]

Although western blot analysis or an enzyme-linked immunosorbent assay (ELISA) test was developed to detect auto antibodies, these tests do not distinguish functional histamine-releasing antibodies from nonfunctional antibodies.[10] These time consuming tests are only performed in a few specialist laboratories and are not available in our department or in majority of the dermatological clinics. It has already been reported that the ASST with positive test being defined as one with serum induced wheals, which is both erythematous and has a diameter of 1.5 mm greater than saline response at 30 minutes is a reasonably predictive clinical test to reveal functional circulating antibodies with a sensitivity of 65-71% and specificity 78-81%.[7] Thus we relied on ASST, the only available and practical test, to demonstrate those patients with auto antibodies.

In our study a higher incidence *H. pylori* antibodies were detected in ASST positive patients in comparison to ASST negative patients. It is postulated that *H. pylori* infection may induce development of pathogenic auto antibodies by molecular mimicry.[11,12] Appelmelk *et al*.[11,12] first demonstrated the molecular mimicry between *H. pylori* and lipopolysaccharide (LPS) anti Lewis antibodies in autoimmune type-B gastritis. Four per cent of patients with a positive ASST showed antinuclear antibodies and two of these patients on further evaluation were diagnosed to have SLE.

Thyroid antibodies were observed in ASST positive patients who showed a moderately significant *P* value, but any clinical abnormalities at the time of examination were not displayed. Isolated association with insulin dependent diabetes mellitus was observed in two ASST positive patients.

In this study lesions lasting for a significantly longer duration and frequency correlated to a higher incidence in ASST positive patients as compared to ASST negative patients.

A study by Sabroe *et al*. concludes that patients with auto antibodies show frequent attacks.[9] There is a statistically significant difference in TSH, thyroid antibodies and ANA between the ASST positive and negative groups, indicating a correlation between a positive ASST and auto immunity. This implies that markers of autoimmunity may be found in many types of chronic urticaria, but ASST is the only clinically demonstrable evidence of autoimmunity. ASST positives had higher IgE titers. This may indicate induction of autoimmunity through cross reactivity or other mechanisms in a population prone to immunological hyper reactivity. Autoimmune disease like thyroid disease, vitilligo, diabetes mellitus, pernicious anemia and rheumatoid arthritis were reported more commonly in patients with autoimmune urticaria.[13] Patients with autoimmune urticaria have no distinctive, diagnostic clinical or histopathological features which differentiate it from non autoimmune cases, although they tend to have more severe urticaria.[2]

In our setting, ASST is the only available test for diagnosis of auto immune urticaria. It is simple, inexpensive, semi-invasive and an easy to perform test which can be done and recorded by the dermatologist. Since conventional approaches of management are frequently unsuccessful, the ASST is especially important from the therapeutic point of view, as it can help the dermatologist to commit himself to initiate immunosuppressive therapies in such patients.

In our study 38% show infective focus on routine screening investigation, CU frequently flared by intercurrent infection. This is due to non-specific effect of circulating pro-inflammatory cytokines or chemokines, either on mast cells or leading to the expression of adhesion molecules on endothelial cells.[14] In only 4-35% of such affected patients did cure of infections lead to improvement of urticaria,[15]

On medical and economic grounds it appears prudent to perform comprehensive laboratory diagnostics only to exclude a particular; individually suspected causative factor and in routine cases, limit the laboratory work up.

Urticaria followed by non steroidal anti inflammatory drug (NSAID) was observed in four patients and one patient presented with urticaria followed by intake of oral contraceptives pills. Withdrawal of these medicines helped to improve of urticaria with in a week. Many drugs can induce urticaria mainly penicillins, salicylates and NSAIDs by non-immunological mast cell activation with a variety of substances including neuropeptides, such as substance P.[15] Neuropeptides elicit histamine but not prostaglandin and leukotriene release. Similarly by inhibiting the cyclo-oxygenase pathway of arachidonic acid metabolism, diverting it to proinflammatory lipoxygenase pathways products and reducing PGE_2_, which is inhibitory for immunological mast cell degranulation.[16] Asprin is the most common drug causing urticaria[16] and accounts for 30-60% of all NSAIDs implicated.[17]

In our study both cholinergic urticaria and dermographism account for 4.7%, which are most common physical urticaria. This corresponds to the study done by Buss *et al*. Cholinergic urticaria occurs due to stimulation of sweating, whether induced by a rise in core temperature, emotion or gustatory stimuli.[18] It is thought to be related to stimulation of cholinergic postganglionic sympathetic nerve supply to sweat glands, itchy wheals appear within minutes of exertion with small 1-3 mm flares that persist for a few minutes to an hour. Dermographism is due to triple response consisting of a local erythema due to capillary vasodilatation, followed by edema and a surrounding flare due to axon reflex induced dilatation of arterioles. This reaction is normal but in 5% of the normal population this physiological response is sufficiently exaggerated to warrant a medical consultation.

Cold urticaria was diagnosed in 2%, corresponding to reports in literature of about 1-3%. It is due to release of histamine, PGD2,[19] leukotrienes, platelet activating factor,[20] TNFα, autoantibodies like IgE and IgM has been recorded.[21] Delayed pressure urticaria was diagnosed in 2% as in the literature and is estimated to be between 2-5% and is due to the release of chemo attractant factors with the up-regulation of vascular adhesion molecules, E-selectin and VCAM 1.[22]

Skin prick test is the most convenient and least expensive method of allergy testing and result can be made available with in 60 minutes. Prick testing helps to trace out type 1 (immunoglobulin E) mediated hypersensitivity specifically.[23,24] Patients with idiopathic urticaria, who were willing and had a high degree of suspicion towards particular food items or aero allergens, an in whom all other clinical and laboratory findings were non contributory, underwent skin prick testing. Standardized extracts for many antigens are readily available. These tests are generally well tolerated with mild erythema and edema that usually subsides within one to two hours. More severe swelling is treated with oral antihistamines, topical steroids and ice-packs.

A positive skin test indicates that the subject is allergic to the particular substance. Bigger papules usually indicate a greater degree of sensitivity. The accuracy of skin tests can vary. Patients react differently to the same test performed at different times or may react positively to an allergen during the test but may not react to it in everyday life.[25] In general, skin tests are most reliable for diagnosing allergies to airborne substances, such as pollen, epithelia, and dust mites.

Diagnosing food allergies can be complex, and may need additional tests or procedures. In our study maximum number of patients reacted positively to brinjal out of the food allergens but due to the small case numbers for prick test, no conclusion can be made. In pollen, maximum number of patients reacted positively to parthenium which is the most common aeroallergen in India.

## Conclusion

Urticaria affects 15-25% of people at least once in their lifetime. It is a frustrating condition for both patient and physician. It is more common in the reproductive age group with a female predominance. In our study, lesions lasted for a significantly longer duration and a higher frequency of attacks in ASST positive patients. Markers of autoimmunity may be found in many types of chronic urticaria, but ASST is the only clinically demonstrable evidence of autoimmunity. ASST provides an easy inexpensive investigation in chronic urticaria and helps direct attention towards the underlying systemic auto immune diseases. It also provides evidence for the rational use of immunomodulators to modify the course of chronic urticaria. Exhaustive work ups with extensive laboratory, technical diagnostics, challenge tests and skin prick testing should be reserved for individual cases following detailed history.

## References

[1] Sheldon JM, Mathews KP, Lovell RG (1954). The vexing urticaria problem; present concepts of etiology and management. J Allergy.

[2] Buss YA, Garrelfs UC, Sticherling M (2007). Chronic urticaria which clinical parameters are pathogenetically relevant? A retrospective investigation of patients. J Dtsch Dermatol Ges.

[3] Grattan CE, Sabroe RA, Greaves MW (2002). Chronic urticaria. J Am Acad Dermatol.

[4] Godse KV (2004). Autologous serum skin test in chronic idiopathic urticaria. J Dermatol Venereol Leprol.

[5] Greaves CE, Sabroe RA, Greaves MW (2002). Chronic urticaria. J AM Acad Dermatol.

[6] Sabroe RA, Grattan CE, Francis DM, Barr RM, Kobza Black A, Greaves MW (1999). The autologous serum skin test: Screening test for auto antibodies in chronic idiopathic urticaria. Br J Dermatol.

[7] George M, Balachandran C, Prabhu S (2008). Chronic idiopathic urticaria: Comparison of clinical feature with positive autologous serum skin test, KMC. Manipal. J Dermatol Venereol Leprol.

[8] Uppal M, Srinivas CR (2004). Wheat induced Urticaria. J Dermatol Venereol Leprol.

[9] Sabroe RA, Seed PT, Francis DM, Barr RM, Black AK, Greaves MW (1999). Chronic idiopathic urticaria: comparison of the clinical features of patients with and without anti FCεR_1_ or anti IgE auto antibodies. J Am Acad Dermatol.

[10] Greaves MW (2000). Chronic Urticaria. J Allergy Clin Immunol.

[11] Ozkaya-Bayazit E, Demir K, Ozgöroğlu E, Kaymakoğlu S, Ozarmağan G (1998). *H. pylori* eradication in patients with chronic urticaria. Arch Dermatol.

[12] Appelmelk BJ, Simoons-Smit I, Negrini R, Moran AP, Aspinall GO, Forte JG (1996). Potential role of molecular mimicry between *H. pylori* lipopolysaccharide and host blood group antigen in autoimmunity. Infect Immun.

[13] Sabroe RA, Seed PT, Francis DM, Barr RM, Black AK, Greaves MW (1999). Chronic idiopathic urticaria: Comparison of the clinical features of patients with and without anti FCεR_1_ or anti IgE auto antibodies. J Am Acad Dermatol.

[14] Tanphaichitr K (1981). Chronic urticaria association with bacterial infection – A case of dental infection. Cutis.

[15] Grattan CE (2003). Asprin sensitivity and urticaria. Clin Exp Dermatol.

[16] Heavey DJ, Kobza-Black A, Barrow SE, Chappell CG, Greaves MW, Dollery CT (1986). Prostaglandin D2 and histamine release in cold urticaria. J Allergy Clin Immunol.

[17] Stevenson DD (1984). Diagnosis, prevention and treatment of adverse reactions to aspirin and non steroidal anti inflammatory drugs. J Allergy Clini Immunol.

[18] Grant RT, Bruce Pearson RS, Comeau WJ (1936). Observations on urticaria provoked by emotion, by exercise and by warming the body. Clin Sci.

[19] Maltby NH, Ind PW, Causon RC, Fuller RW, Taylor GW (1989). Leukotriene E 4 releases in cold urticaria. Clin Exp Allergy.

[20] Grandel KE, Farr RS, Wanderer AA, Eisenstadt TC, Wasserman SI (1985). Association of platelets-activating factor with primary acquired cold urticaria. N Engl Med.

[21] Wanderer AA, Maselli R, Ellis EF, Ishizaka K (1971). Immunological characterization of serum factors responsible for cold urticaria. J Allergy Clin Immunol.

[22] Czarnetzki BM, Meentken J, Kolde G, Bröcker EB (1985). Morphology of cellular infiltrate in delayed pressure urticaria. J Am Acad Dermatol.

[23] Uppal M, Srinivas CR (2004). Wheat induced Urticaria. Indian J Dermatol Venereol Leprol.

[24] Mahesh PA, Kushalappa PA, Holla AD, Vedanthan PK (2005). House dust mite sensitivity is a factor in chronic urticaria. Indian J Dermatol Venereol Leprol.

[25] Chemboli L, Srinivas CR (2007). Type 1 hypersensitivity to parthenium hysterophorus in patients with parthenium dermatitis. Indian J Dermatol Venereol Leprol.

